# *SERPINA1* and More? A Putative Genetic Contributor to Pulmonary Dysfunction in Alpha-1 Antitrypsin Deficiency

**DOI:** 10.3390/jcm12051708

**Published:** 2023-02-21

**Authors:** Aleksandra Jezela-Stanek, Joanna Chorostowska-Wynimko

**Affiliations:** Department of Genetics and Clinical Immunology, National Institute of Tuberculosis and Lung Diseases, 01-138 Warsaw, Poland

**Keywords:** AATD, mRNA, methylation, SNP, micro-RNA, *SERPINA1* gene

## Abstract

Alpha-1 antitrypsin deficiency (AATD) is a common inherited disorder associated with an increased risk of pulmonary disease. Its clinical presentation, including the nature and severity of organ involvement, is highly variable and unpredictable and is not as strongly linked to genotype and environmental exposure (e.g., smoking history) as might be expected. Significant differences were observed within matched populations of severe AATD patients regarding risk of complications, age at onset, and disease course, including the dynamics of lung function decline. Genetic factors are among the putative modifiers contributing to the clinical variability in AATD, yet their role remains elusive. Here, we review and summarise our current understanding of epigenetic and genetic modifiers of pulmonary dysfunction in subjects with AATD.

## 1. Introduction

No protein fits a simple gene–product relationship; the progression from DNA sequence to polypeptide generation is complex, with several vital processes such as posttranslational modifications involved. However, protein functionality also depends on many other factors, pathways, and/or modifiers, which influence the complexity of the clinical course of many inherited diseases. Alpha-1 antitrypsin deficiency (AATD) is an excellent example of such interplay with the highly polymorphic *SERPINA1* gene product, which, similar to other serpins, is prone to conformational shifts and therefore significant changes in its functionality [1,2].

AATD (ORPHA 60, MIM #613490) is one of the most common inherited rare diseases in Caucasians, with a prevalence of 1–5/10,000 [3]. Its clinical presentation varies according to the affected organ and severity. Typically, AATD manifests in adults with respiratory disorders, most often early-onset emphysema and/or bronchiectasis, or as a liver pathology of persistent jaundice or cirrhosis in all age groups, newborns, children, and adults. Panniculitis and vasculitis, which have also been linked to AATD, are significantly less prevalent. 

The natural history of organ-specific pathologies related to AATD is highly unpredictable, with few identified risk factors, such as smoking or occupational exposure to lung or alcohol abuse or viral hepatitis. This is also true for the prognosis and rate of disease progression, as exemplified by a decline in lung function or an increase in liver enzymes. While significant progress has been made in understanding the mechanisms underlying AATD, there is more to be learned [4]. A protease–antiprotease imbalance is hypothesised to be the primary cause of lung destruction; therefore, emphysema does not fully explain the wide variety of clinical phenotypes in AATD. There is increasing evidence suggesting that, in addition to *SERPINA1* alterations, other genetic factors and modifiers play an important role [5]. In the era of genomic medicine, widespread use of molecular diagnostics, and high-throughput technologies, the identification of such factors is of primary clinical interest. This review discusses our current understanding of genetic modifiers and prognostic markers contributing to the different respiratory phenotypes of AATD.

## 2. Genetic Variants and Polymorphisms

AAT coding gene *SERPINA1* (* 107400; SERPIN PEPTIDASE INHIBITOR, CLADE A, MEMBER 1) was previously known as a protease inhibitor (PI). Its genomic length is 10.2 kb with a 1434-bp coding region [6]. The gene has 4 introns: exon 1, the 5-prime portion of exon 2, and the 3-prime portion of exon 5, which are noncoding regions. The first intron, 5.3 kb long, contains a 143-amino acid open reading frame (which does not appear to be an actual protein coding region), an Alu family sequence, and a pseudo transcription initiation region. Hepatocytes are the major source of AAT, but the gene is also expressed in mononuclear phagocytes and neutrophils [7].

Numerous genetic factors have known or possible direct effects on the clinical appearance of AATD, including single nucleotide polymorphisms (SNPs), DNA methylation, altered microRNA (miRNA) expression, and SERPINA1 mRNA isoforms (Table 1). Other inherited variants may indirectly and independently of AATD affect individual susceptibility to progressive airway obstruction, as shown by the rate of decline in lung density or function. This is the case with IREB2, which encodes iron-responsive element-binding protein 2 [8], a group-specific component that is the major vitamin D-binding protein [9], as well as a variable number of tandem repeats within the interleukin-1 receptor antagonist gene or the tumour necrosis factor (TNF)-α 308 G/A variant in the Asian population [10], which have been shown to advance the development of chronic obstructive pulmonary disease (COPD).

### 2.1. SNPs (Single Nucleotide Polymorphisms)

A SNP is the most common type of genetic variation. By definition, a specific variant is identified as a SNP if it is observed in ≥1% of the population and the affected gene has more than one allele. As presented in Table 1, clinically significant SNPs identified in AATD include variations in NOS3, GSTP1, TNF-α, IL10, CHRNA3, IREB2, mEH, and immune-related genes.

#### 2.1.1. NOS3

Endothelial nitric oxide synthase (NOS3) oxidatively deaminates L-arginine to L-citrulline, releasing nitric oxide. In turn, NOS3 regulates vascular and airway tone in the lungs and influences various aspects of airway homeostasis [11]. Six polymorphic sites within the *NOS3* gene (924A/G, 788C/T, 691C/T, 774C/T, 894G/T, and 1998C/G) in 345 AATD patients (PI*Z homozygotes or compound heterozygotes of PI*Z with other deficiency alleles) and 93 control individuals were analysed. A detailed clinical and smoking history in AATD individuals have been described elsewhere [12,13]. A higher incidence of the 774T and 894T alleles was observed in more severely affected individuals compared with less severe cases (0.417 vs. 0.289 and 0.427 vs. 0.344, respectively), suggesting a link between *NOS3* allelic variants and the pathogenesis and/or severity of the disease. 

Novoradovsky et al. analysed smoking status in 55 patients with a predicted forced expiratory volume in 1 s [FEV1] that was < 35% of the predicted normal value [FEV1%pred] and in matched controls [11]. They observed a trend toward significance in the smokers plus ex-smokers in the frequency of 774T and 894T alleles. Specifically, in smokers and ex-smokers, the 774T allele frequencies were 0.460 and 0.275 (*p* = 0.072), whereas those of the 894T allele were 0.480 and 0.300 (*p* = 0.083), respectively. In non-smokers, the 774T allele frequencies were 0.365 and 0.278 (*p* = 0.371), and the 894T allele frequencies were 0.385 and 0.328 (*p* = 0.527) in patients and controls, respectively. Given that almost all affected individuals and controls were ex-smokers or non-smokers, investigating the effect of current smoking has not been possible.

#### 2.1.2. GSTP1

Glutathione S-transferase P1 is involved in the detoxification of electrophilic substances present in tobacco smoke. Therefore, it has been suggested to play a role in the pathogenesis of smoking-related respiratory disorders [14]. The *GSTP1* 105Val polymorphism, which results in reduced enzyme activity in vitro, has been of particular interest. Rodriguez et al. [14] evaluated the frequency of Ile105Val polymorphisms in the general population compared with patients with AATD or smoking-related COPD. A total of 99 patients with COPD (current or ex-smokers), 69 patients with AATD with a wide range of lung function impairments, and 198 healthy volunteers were included in the analyses.

The *GSTP1* 105Val frequency was significantly increased in the AATD group (vs. healthy controls: odds ratio [OR] 2.09, 95% confidence interval [CI] 1.17–3.72; vs. COPD group: OR 2.41, 95% CI 1.27–4.59) but was comparable between COPD patients and healthy controls. This result was not observed in COPD patients with normal AAT levels; lung function was not significantly different according to the *GSTP1* genotype. Interestingly, lung function (FEV1%pred) was significantly impaired in AATD carriers of *GSTP1* 105Val. This phenomenon was not observed in PI*MM COPD patients or healthy controls.

#### 2.1.3. TNF-α/TNF (TNFA) Gene

Tumor necrosis factor (TNF) is a multifunctional proinflammatory cytokine secreted predominantly by monocytes/macrophages that has effects on lipid metabolism, coagulation, insulin resistance, and endothelial function [15]. 

The rs361525 variant (G-238A) of *TNF-α* was linked to a higher incidence of chronic bronchitis in AATD in 424 unrelated PI*ZZ subjects with a known history of chronic bronchitis, lung function impairment as assessed by the FEV1%pred, and emphysema and bronchiectasis confirmed by high-resolution CT scanning [16]. 

Higher levels of the pro-inflammatory cytokine TNF-α have been repeatedly demonstrated in sputum, bronchial biopsy, and peripheral blood samples in both stable and exacerbated COPD patients [17,18,19]. This specific genotype was found to be associated with higher TNF-α expression in lung secretions. It increased downstream signalling in vivo and bioactivity in vitro, suggesting a link to the COPD disease phenotype and progression [20]. Thus, Wood et al. [16] linked *TNFα* variants to AATD clinical phenotypes. These phenotypes were determined based on full clinical assessments, including smoke exposure, the presence of chronic bronchitis, lung function testing, and high-resolution chest CT scanning. A significant difference in the rs361525 genotype (*p* = 0.01) and allele (*p* = 0.01) frequency was observed between subjects with and those without chronic bronchitis, independent of the presence of other clinical phenotypes. No correlation with the TNF-α plasma level was observed.

#### 2.1.4. Interleukin 10

DeMeo et al. [21] conducted family-based association analyses in a group of 378 PI*ZZ individuals from 167 families. They hypothesised that genetic determinants of obstructive lung disease might be modifiers of airflow obstruction in individuals with severe AATD. A panel of 10 genes (*IL10*, *TNF*, *GSTP1*, *NOS1*, *NOS3*, *SERPINA3*, *SERPINE2*, *SFTPB*, *TGFB1*, and *EPHX1*) previously linked to asthma and/or COPD was analysed. Genetic analysis was performed in all 428 AATD subjects (including genotypes from 50 parents), while spirometry was performed in 378 affected PI*ZZ individuals, which allowed the determination of genetic transmission within families. The qualitative phenotype of COPD was defined using post-bronchodilator spirometry values (FEV1 and the ratio of FEV1/forced vital capacity [FVC]), and a qualitative phenotype of moderate-to-severe COPD was defined for individuals with a predicted FEV1 < 50%pred. Consistent findings for both quantitative and qualitative airflow obstruction phenotypes were noted for *IL10* SNPs only. Further assessments with a sliding window haplotype analysis using the 11 SNPs in *IL10*, as well as 8, 4, 3, and 2 adjacent SNP sliding windows, revealed an association of the −1082 A/G promoter polymorphism with lower lung function but an association of the mutant G (minor) allele with higher lung function. The 1082 rs1800896 SNP has been associated with a functional effect on the IL-10 protein level: the A wild-type allele is associated with a lower IL-10 level, and the G mutant allele with a higher IL-10 level.

#### 2.1.5. CHRNA3 and IREB2 Genes

Prior reports of genome-wide association studies (GWAS) of COPD showed significant associations between COPD susceptibility and SNPs in the 15q region [22], as well as associations with the presence and severity of emphysema [23]. The association between specific SNPs in the chromosome 15q region encompassing *CHRNA3* and *IREB2* was studied in 378 PI*ZZ individuals in the AAT Genetic Modifiers Study and a replication cohort of 458 subjects from the UK AATD national registry [24]. The authors hypothesised that SNPs in this chromosomal region might be modifiers of intermediate phenotypes of COPD in subjects with severe AATD. Importantly, for COPD phenotyping, both lung function and lung morphology assessed by CT scanning were used to overcome some of the heterogeneity inherent in pulmonary function classifications based solely on spirometric measurements.

In contrast to the UK AATD national registry, the AAT Genetic Modifiers Study has shown that SNPs in the genes *IREB2*, *LOC123688*, and *CHRNA3* are associated with specific lung function phenotypes in AATD PI*ZZ subjects. Specifically, rs2568494 in *IREB2*, rs8034191 in *LOC123688*, and rs1051730 in *CHRNA3* were associated with pre-bronchodilator FEV1%pred (*p* < 0.05). Two of these SNPs (rs2568494 and rs1051730) were linked to post-bronchodilator FEV1 %pred (postFEV1%pred) and the preFEV1/FVC ratio; rs1051730 was also linked to the post-bronchodilator FEV1/FVC ratio. There was no association between any of the genotyped SNPs and pack–years of smoking. There is some evidence that the modifier effects of *IREB2* and *CHRNA3* may be more prominent in males (rs2568494, rs8034191, and rs1051730 for post-bronchodilator FEV1/FVC), which may support the existence of sex-specific features of COPD susceptibility and severity observed in PI*ZZ patients [25].

#### 2.1.6. Immune-Related Genes

The phenotypic manifestation of AATD may be influenced by incomplete penetrance and/or variable expression of genes other than pathogenic *SERPINA1* alleles. Rigobello et al. performed whole-exome sequencing (WES) in a group of siblings (*n* = 9) from four different families with extreme phenotypes of the disease. The family members were concordant for genotype but discordant for clinical presentation, i.e., at least one individual was suffering from emphysema, while the other was not affected, and identified variants were compared across unrelated families [26]. By restricting the analyses to AATD siblings with extreme phenotypes, researchers limited the effect of heterogeneity to allow more reliable identification of factors contributing to the development of emphysema. In contrast to other WES studies, no exclusion of common variants was performed as per the justified assumption that frequent alleles may modify the effects of some rare alleles.

As a result, 41,877 functionally annotated variants were identified, including 20,748 (49.5%) synonymous and 21,129 (50.5%) non-synonymous variants. There were 15 variants of 14 genes in the recessive model (57% immune-related) and 23 variants of 21 genes in the dominant model (29% immune-related) in the affected but not non-affected AATD individuals. All variants were identified by pathway analysis as functionally important in innate and adaptive immunity and were primarily involved in the activation of the complement cascade, antigen presentation, and immune response regulation, e.g., the rs3747517 variant of *IFIH1* and variants of *AKNA* and *MIB2*. Of note, in the group of non-affected individuals, other genetic variants with a known immune suppressor function in innate and adaptive immunity, including HLA-C, HLA-DQB1, and HLA-DRB1 variants, were detected.

Other gene variants involved in regulating immune homeostasis and maintaining self-tolerance were identified as predisposing to or protecting from emphysema in siblings with AATD. In affected individuals, these were mainly genes with immune-activating functions; in non-affected individuals, immune-suppressing gene variants involving antigen processing, MHC-I presentation, and TCR and PD-1 signalling were present. Interestingly, some of the genes identified in symptomatic patients, including *PPT2*, *DNTTIP2*, *IQCG*, and *PRDM16*, had also been reported earlier in GWAS or transcriptomic analyses in patients with COPD [27], highlighting their potential significance for COPD predisposition. The dynein regulatory complex protein 9, encoded by the *IQCG* gene, is active only in the respiratory system [28], where it is incorporated in the nexin–dynein regulatory complex, a key regulator of ciliary/flagellar motility that maintains the alignment and integrity of the distal axoneme and regulates microtubule sliding in motile axonemes. Consequently, the direct causative roles of gene variants identified in COPD pathogenesis remain unclear.

By precise phenotypic matching, Rigobello et al. provided objective evidence of SNPs distributed solely in affected or non-affected siblings with AATD; the distribution differed substantially between the two groups. Symptomatic patients harboured, for example, the rs3747517 variant of the interferon-induced helicase C domain-containing protein 1 (*IFIH1*) gene, which encodes a cytoplasmic viral RNA receptor that activates the type I interferon signalling adaptor molecule via the mitochondrial antiviral signalling protein. Rice et al. [29] showed that gain-of-function variants in *IFIH1* are linked to several human disease phenotypes associated with upregulated type I interferon signalling. 

### 2.2. Changes in DNA Methylation

DNA methylation, an epigenetic mechanism that involves the transfer of a methyl group to the C5 position of cytosine to form 5-methylcytosine, affects gene expression. Epigenetic regulation has been hypothesised to contribute to COPD development, as the development of this disease cannot be fully explained exclusively by inherited factors, i.e., DNA.

Qiu et al. were the first to conduct a comprehensive assessment of DNA methylation to analyse its role as a regulator of gene transcription in the context of lung function impairment and COPD [30]. Analyses were performed independently in two cohorts, both with/without COPD (or unclassified COPD status): 369 subjects from the Boston Early-Onset COPD Study (with/without a history of cigarette smoking) and 1085 patients from the International COPD Genetics Network with a history of cigarette smoking. 

This array-based methylation analysis encompassed 27,578 CpG sites in 14,475 consensus coding sequences and identified two CpG sites (cg02181506 and cg24621042) in the *SERPINA1* gene as the highest-ranking methylation marks associated with COPD. Results were similar in both cohorts and were independent of COPD severity and cigarette exposure. Possible associations of DNA methylation with lung function parameters were also investigated. A total of 4798 and 4899 CpG sites were significantly associated with the FEV1/FVC ratio and FEV1, respectively. Interestingly, no participant was a carrier of biallelic variants in *SERPINA1.* Thus, *SERPINA1* hypomethylation was proposed as an essential risk factor for COPD and bronchial obstruction.

Sundar et al. analysed DNA methylation in parenchymal lung tissue [31] and sought to determine whether the genome-wide lung DNA methylation profile of smokers (27.14 ± 5.96 pack-years for 7/8 individuals) and patients with COPD (28.8 ± 4.32 pack-years for 7/8 patients) differed significantly compared with non-smokers. Information on AATD status was not provided in this study. Based on previous data showing that *SERPINA1* is significantly hypomethylated in smokers and patients with COPD, *SERPINA1* (cg02181506 site) was selected for gene-specific CpG assessment. Contrary to the results of Qiu et al. [30], *SERPINA1* hypomethylation in smokers and patients with COPD did not show significant changes. A correlation in the *SERPINA1* methylation status (as methylation percentages) among non-smoker groups was noted, while no significant hypomethylation of the *SERPINA1* CpG site in smokers and COPD groups compared with non-smokers was evident.

Beckmeyer-Borowko et al. [32] evaluated *SERPINA1* methylation as a possible determinant of lung function and its decline over the life course in a tobacco smoke-exposed population. The objective was to analyse the associations of a comprehensive set of methylation sites in the *SERPINA1* gene cluster with lung function parameters and 10- to 15-year lung function decline. The analysed CpGs within the *SERPINA* gene cluster were from 12 genes: *PPP4R4*, *SERPINA10*, *SERPINA6*, *SERPINA1*, *SERPINA11*, *SERPINA9*, *SERPINA12*, *SERPINA4*, *SERPINA5*, *SERPINA3*, *SERPINA13*, and *GSC*. Overall, 1076 adult ever-smokers from three population-based European cohorts (SAPALDIA, ECRHS, and NFBC) and 259 tobacco smoke-exposed children from the ALSPAC cohort were analysed. None of the methylation sites in the *SERPINA1* gene showed any association with lung function after multiple testing corrections both in multivariate cross-sectional and longitudinal regression analyses. On the other hand, methylation at cg08257009, located 32 kb downstream from *SERPINA1*, was significantly linked to the FEV1/FVC ratio in adults but not children. Interestingly, relative hypermethylation, but not hypomethylation, at the *SERPINA1* loci cg02181506 and cg24621042, as shown by Qiu et al. [30], was associated with a lower concentration of serum ATT in the SAPALDIA cohort.

Recently, Rotondo et al. [33] showed evidence of the significance of *SERPINA1* methylation for COPD risk in acute coronary syndrome (ACS) patients. The methylation analysis was carried out in 115 ACS patients, including 30 COPD+ and 85 COPD- according to lung function, based on spirometry. Mean age  ± standard deviation [SD] was 65  ±  9 years, the inclusion criteria comprised smokers or former smokers (≥10 pack/years) and a clinical diagnosis of ACS, while the exclusion criteria included a previous diagnosis of COPD and/or asthma, known pulmonary diseases other than COPD, ongoing pneumonia, ongoing heart failure, documented or suspicion of malignant disease, life expectancy  <  1 year, and recent thoracic trauma. *SERPINA1* was hypermethylated in 24/30 (80%) COPD+ and 48/85 (56.5%) COPD- (*p* < 0.05). The authors concluded that hypermethylation of *SERPINA1* may represent a potential biomarker for predicting COPD development in acute coronary syndrome patients.

### 2.3. miRNAs

Endoplasmic reticulum (ER) stress, resulting from disturbances in ER homeostasis, leads to the activation of several signalling pathways, including the unfolded protein response (UPR). This process was also described in AATD as the ‘AAT Z variant’ [34,35], which represents a substitution of glutamic acid with lysine at position 342 of the mature protein (Glu342Lys), which is misfolded and undergoes intracellular polymerisation in the ER, activating the UPR. Disturbances in miRNA expression were shown to be involved in this process by direct regulation of UPR components or effectors [36]. 

Hassan et al. assessed miR-199a-5p as a potential clinically relevant biomarker of AATD symptomology [37]. The researchers investigated ex vivo miRNA expression and function in monocytes from asymptomatic PI*MM non-smokers (*n* = 8) and PI*ZZ (*n* = 11) individuals and patients with COPD (7 PI*MM and 11 PI*ZZ) to identify miRNA(s) regulating the UPR. miR-199a-5p was the most upregulated miRNA in asymptomatic PI*ZZ individuals compared with asymptomatic PI*MM individuals, and the UPR markers GRP78, p50, and p65 were overexpressed in monocytes from asymptomatic but not symptomatic ZZ individuals. Moreover, in MM monocytes, in vitro pharmacological induction of ER stress led to increased miR-199a-5p expression, followed by increased DNA methylation at CpG sites upstream of miR-199a-5p and, thereby, silencing of miR-199a-5p. 

Putative targets of miR-199a-5p enriched in the ER protein folding pathway that modulate the expression of UPR components were further defined using bioinformatic tools. miR-199a-5p was found to modulate the expression of UPR components directly. That study was the first to present evidence of higher expression of key UPR components in monocytes from symptomatic ZZ individuals compared with asymptomatic individuals and of lower miR-199a-5p expression in MM and ZZ monocytes from COPD patients compared with asymptomatic MM or ZZ individuals. Although these results have helped further our understanding of the UPR in ER stress-related diseases such as COPD in AATD, their clinical significance requires further investigation.

Considering that members of the miR-320 family have potential specific binding sites in the 3′ untranslated region (UTR) of the *SERPINA1* gene, Matamala et al. showed increased miR-320c expression in 98 individuals with pulmonary disease irrespectively of the AAT serum level [38]. Likewise, they demonstrated that miR-320c expression was elevated in vitro in HL60 cells exposed to an inflammatory milieu as well as in response to the pro-inflammatory factor lipopolysaccharide. These results suggest a significant role for miR-320c in AATD as an indicator of inflammatory processes in pulmonary diseases; its potential biomarker significance remains to be determined.

Esquinas et al. investigated differences in mRNA and miRNA expression and their possible association with the severity of COPD in PI*ZZ AATD [39]. Gene expression profiling of peripheral blood mononuclear cells from six mild and six severe PI*ZZ COPD patients revealed 205 differentially expressed genes as well as 28 differentially expressed miRNAs. Specifically, hsa-miR-335-5p was downregulated, while 12 target genes involved in cytokine, MAPK/mk2, and JNK signalling, as well as angiogenesis, were upregulated in severe compared with mild patients. Altogether, these results suggest the role of miRNAs in COPD progression and provide evidence of molecular pathways affecting immune cell activity. 

### 2.4. SERPINA1 mRNA Isoforms

The *SERPINA1* gene harbours over 100 polymorphisms, as summarised in the Human Gene Mutation Database [40]. While not all are pathogenic, their potential biological significance has not been verified. The *SERPINA1* gene is also worthy of attention due to the complexity of its transcripts, which encompass 11 mRNA isoforms with two transcription start sites, six splicing donors, and three acceptor sites. The different transcripts are generated by alternative splicing in the 5′-UTR [41]. Since the efficiency of the transcript-specific translation may play an important role in tissue-specific AAT expression, the role of the distinct 5′-UTR in a posttranslational regulatory program, resulting in differences in mRNA expression, was assessed by Corley et al. [41]. They proposed that noncoding SERPINA1 fragments control this program via the interplay between alternative splicing and translation efficiency mediated by upstream open reading frames (uORFs) and RNA structure. uORFs are located within the 5′ leader mRNA sequence and are considered inhibitors of downstream translation initiation of protein-coding sequences.

From a practical point of view, the proposed model offers a stimulating contribution to our knowledge of tissue-specific AAT expression, which may provide novel insight into potential therapeutic interventions to increase lung-specific AAT concentrations in affected individuals. The therapeutic strategy may, for example, involve antisense oligonucleotides targeting Kozak sequences (CRCCaugG) near the start codon, which influence the rate of translation initiation [42] around uORFs in *SERPINA1* transcripts. 

### 2.5. Long Non-Coding RNAs (lncRNAs)

Recently, the first (preliminary) data on the changes in lncRNA expression during augmentation therapy in AATD patients have been published [43]. In peripheral blood monocytes (PBMs) isolated from *n* = 5 ZZ individuals pre- and post (day 2)-AAT augmentation therapy, lncRNA microarray profiling was performed. In total, 17.761 lncRNAs were detectable across all samples, which allowed for the identification of 7509 lncRNAs with differential expression post-augmentation therapy: 3084—increased and 4425—decreased (fold change ≥ 2).

Since the results refer to the treatment (supporting the manifold effects of AAT augmentation therapy) and have no obvious relation to the AATD pulmonary manifestations, they are not listed in the Table 1.

**Table 1:** Putative genetic contributors to pulmonary decline in AATD and/or *SERPINA1*.

Single Nucleotide Polymorphism (SNP)			
*NOS3*	endothelial nitric oxide synthase	statistically significant association between the polymorphic sites 774C/T and 894G/T and severity of lung disease in individuals with AAT deficiency	Novoradovsky et al., 1999 [11]
*GSTP1*	glutathione s-transferase P1	the frequency of the 105Val polymorphism is increased in patients with AAT deficiency; the modulatory role has been observed only in smokers	Rodriguez et al., 2005 [14]
*TNFA*	tumour necrosis factor alpha	association between the A allele of rs361525 subjects and chronic bronchitis	Wood et al., 2008 [16]
*IL10*	interleukin 10	*IL10* rs1800871 and rs1518110 are likely important modifiers for the development of COPD in individuals with severe AAT deficiency	Demeo et al., 2008 [21]
*CHRNA3*	cholinergic nicotine receptor alpha3	*IREB2* rs2568494 and *CHRNA3* rs1051730 are potential genetic modifiers of COPD phenotypes in individuals with severe AAT deficiency and may be sex-specific	Kim et al., 2012 [24]
*IREB2*	iron regulatory binding protein 2		
immune genes		possible genetic susceptibility factors for emphysema observed in AATD siblings	Rigobello et al., 2018 [26]
**DNA methylation**			
*SERPINA1*	cg02181506 and cg24621042 relative hypomethylation	significant, replicable associations between *SERPINA1* hypomethylation and COPD and lower average lung function phenotypes	Qiu et al., 2012 [30]
*TGFBI*	cg07852148, relative hypomethylation	associated with ever-smoking after adjustment for age and sex in AATD	Siedlinski et al., 2012 [44]
*SERPINA1*	relative hypomethylation of cg02181506 and cg24621042	associated with lower circulating AAT	Beckmeyer-Borowko et al., 2018 [32]
**microRNA**			
miR-199a-5p	a key regulator of the unfolded protein response in AAT-deficient monocytes (epigenetic silencing of its expression regulates this process in chronic obstructive pulmonary disease)		Hassan et al., 2014 [37]
hsa-miR-335	downregulation of miRNA (hsa-miR-335) and activation of pathways related to inflammation and angiogenesis on comparing patients with severe vs. mild COPD-AATD		Esquinas et al., 2017 [39]
miR-320c	miR-320c inhibited *SERPINA1* expression in a hepatic cell line and its levels in blood were associated with lung disease in a cohort of patients with different AAT serum levels		Matamala et al., 2020 [38]
**mRNA**			
*SERPINA1* mRNAs	RNA structure governs a complex posttranscriptional regulatory program of α-1-antitrypsin expression		Corley et al., 2017 [41]

## 3. Discussion

Clinical experience has shown that the AATD phenotype is only partially attributed to the genotype, i.e., pathogenic variants of *SERPINA1*, and this disparity is not fully explained by smoking or environmental exposure. Moreover, there is high variability in lung disease presentation and course in AATD individuals. As in any inherited condition, incomplete penetrance and variable expressivity should be considered. Nonetheless, it is likely that genes other than *SERPINA1* and genetic polymorphisms contribute to the penetrance and expressivity of AATD. Research is ongoing to identify genetic modifiers and reliable prognostic biomarkers in AATD to facilitate the identification and targeted care of high-risk individuals.

Currently described genetic factors, which may contribute to the pulmonary phenotype of AATD, can be categorised as follows: SNPs, changes in DNA methylation, altered miRNA expression, and RNA structure-mediated posttranscriptional modifications (Table 1). Despite significant research efforts, no definitive candidate marker or mechanism involved in the respiratory phenotypic presentation of AATD has been identified. 

As presented in this review, these analyses have several limitations, such as the size of the cohorts and/or poor matching of the patient groups [36,41]. Many exciting observations will require independent validation in larger cohorts and/or verification within the clinical context. For example, the gene expression profiles reported by Esquinas et al. [39] and Rigobello et al. [26] involved 12 COPD PI*ZZ patients (comparable in age, sex, exacerbations, comorbidities, and use of augmentation therapy) and the clinical data from four families. While small study groups are an accepted norm in rare disease research, prospective clinical data collected from international registries, such as the European EARCO registry for AATD, might provide a more reliable source for analyses. In addition, the sizes of the study groups need to be interpreted in the context of the study design. Rigobello et al. applied a case–control study design by comparing closely related AATD probands with contrasting clinical phenotypes. Consequently, certain genetic variants (SNPs) were found exclusively in patients with AATD-related respiratory disease and not in their non-affected siblings with AATD. 

Data should also be considered in light of their actual, and not just statistical, significance. A statistical cut-off of *p* ≤ 0.05 is considered the gold standard and directly affects protocol design as well as the expected size of the study groups. Clinical significance, or the minimum clinically important difference, which allocates the specific needs of rare disease research without breaching clearly stated assumptions and precise research methodology, is becoming more accepted. The ultimate verification of both data and conclusions comes from their repeatability in independent, but clinically comparable, cohorts [16]. Wood et al. demonstrated a statistically significant difference in the frequency of *TNF-α* rs361525 between PI*ZZ individuals with and those without chronic bronchitis (genotype, *p* = 0.01; and allele, *p* = 0.01). However, they were unable to verify this observation in a different cohort [4]. DeMeo et al. confirmed this association between *TNF* and bronchial obstruction in AATD patients; however, there was no overlap in the clinically significant *TNF* SNPs, preventing any meaningful conclusions [21]. Considering the immunoregulatory role of the AAT protein, independent of its antineutrophil elastase activity and its interaction with the neutrophil-binding TNF-α receptor [45], the potential role of TNF in AATD is worthy of further investigation. There are other interesting observations in need of verification, such as the increased expression of components of the MAPK signalling pathway, specifically *EREG, EGR3*, and *TRIB1*, which are important for regulating the inflammatory response [39,46] and modifying the AATD clinical phenotype.

There are also contradictory studies on the potential modifying role of certain polymorphisms. DeMeo et al. [21] explored *GSTP1* and *NOS3* polymorphisms in a family-based study and were unable to confirm the association between the *GSTP1* 105Val polymorphism and COPD in AATD, proposed by Rodriguez et al. [14], or the increased frequencies of the *NOS3* 774T and 894T alleles in AATD individuals with severe lung disease, suggested by Novoradovsky et al. [11]. 

The interaction between genetic, epigenetic, and environmental factors and their effect on AATD clinical presentation is convoluted at present. Whether the DNA methylation changes observed in certain AATD phenotypes are of primary or secondary origin, i.e., result from environmental factors, smoking, or inflammatory stimuli, is an ongoing debate [30]. To fully address this issue, tissue-specific, whole-genome, age-dependent research on methylation markers and their effects on specific physiological mechanisms is warranted. In a blood-based methylome study, Beckmeyer-Borowko et al. did not observe significant associations between *SERPINA1* gene methylation changes and lung function parameters after multiple testing corrections [32]. Similarly, a more extensive epigenome-wide association study on smoking showed no relevant link between *SERPINA1* epigenetic signatures and smoking [47]. While some researchers believe that the effects of smoking on *SERPINA1* methylation do not warrant further investigation, the potential bias introduced by environmental factors, including smoking, continues to be an issue in AATD research. Methodological issues also add to the complexity. A potential link between decreased miR-335-5p expression and AATD-related emphysema was independently proposed by Ezzie et al. [48] and Van Pottelberge et al. [49]; however, differences in clinical characteristics and the biological compartments evaluated rendered analysis difficult. Comparing lung samples from COPD and non-COPD smokers [48] and sputum samples from COPD smokers, non-COPD smokers, and never-smokers [49] did not enable reliable interpretation of observed miRNA up- or downregulation. Moreover, as with DNA methylation, the question of a primary or secondary causative link to disease severity, co-morbidities, medications, and external factors, such as smoking, remains unanswered. 

Finally, in addition to environmental stimuli, the potential effects of genetic factors other than *SERPINA1* cannot be ignored. Although their role in clinical phenotype variability in AATD has not been confirmed, numerous candidate modifiers have been suggested, mostly among immune-related pathways [26,39]. Likewise, most, if not all, research studies and clinical guidelines are focused on the phenotypic presentations of the PI*Z and PI*S alleles. There is a shortage of clinical data on the phenotypic manifestation of rare *SERPINA1* variants. Efforts should be made to collect data on the clinical significance of many largely unknown variants.

## Data Availability

Not applicable.
